# An Essay on Superfœtation

**Published:** 1807-06

**Authors:** William Dewees

**Affiliations:** Philadelphia


### Medical and Physical Journal.

VOL. XVII.] JUNE, 1807. [NO. 100.

*Printed for R. PHILLIPS, by W. Thorne, Red Lion Court, Fleet Street, London.*

### *An Essay on Superfœtation.* By DR. WILLIAM DEWEES, *of Philadelphia.*

THE possibility of superfœtation is not a new idea: the present Essay is an attempt to revive it, and to establish its probability, as well by reasoning as by facts. Many cases have occurred since the history of medicine to countenance a belief in it, and it did for a long time prevail; but, like many other opinions that could not admit of absolute demonstration, it has long since been laid aside, or in other words, held as a physical impossibility.

It has been urged that superfœtation could not take place; first, from the indispensible necessity of the male semen passing through the mouth of the uterus to produce conception; and secondly, as soon as this event has taken place, the os uteri closes, and becomes impervious to the semen ejected in subsequent acts of coition.

If these opinions are founded on facts, the impossibility of superfœtation is established beyond the power of controversy; but these are the points to be investigated.

Let us therefore inquire into the probabiiity of this theory, and see how well it will accord with facts and reason.

Before we proceed, however, any further, let us for a moment consider the anatomy and situation of the unimpregnated uterus: we shall find it a small flattened body, floating as it were in the middle of the pelvis; composed of muscular fibres, nerves, blood-vessels, lymphatics, &c. divided in general by anatomists into body, fundus, and neck; having two small perforations near its fundus, which are the passages to and from the Fallopian tubes; with a cavity capable of admitting a bean if its sides were distracted; but these sides are, for the most part, if not always, when not mechanically stretched by some power or other, in a state of collapse or contact, or at least as much so, as a pretty thick mucus with which it is constantly supplied, will admit of; having a neck pendant in the pelvis, pelvis, which is pervious and capable of admitting a probe and this, like the body, is also lined with a thick ropy mucus; the termination of this neck is the os tincæ, which has no fixed place in the pelvis or vagina; it is sometimes found inclining to the right, at others to the left; now looking upwards and anteriorly, presently dipping downwards and posteriorly; but most frequently it is found (especially in women who have borne children) lying or resting on the internal face of the perinæum; possessing no power that we are acquainted with, to fix its situation at any time, consequently is subject to all the changes of place that the pressure of the abdominal muscles may give it, when exerted in making of water and going to stool; to all those that may arise from the weight of the intestines and viscera; from the full or empty bladder; from the distended or flaccid rectum, &c.

After having thus far considered the uterus, let us next attend to what must be effected by the male, that impregnation may take place agreeably to the theory just mentioned. It is, that the male organs of generation must have sufficient vigour to push a thick tenacious fluid thro' the narrow aperture of the neck of the uterus, and make a lodgement of it within its cavity: can it for an instant be supposed they possess this power? we think they do not for the following reasons.

1st. Because they have not, in our opinion, sufficient strength for this purpose, if it even be admitted that the extremity of the male urethra and the os tincæ of the female were in a state of perfect apposition.

2dly. Because they seldom or never are in a state of apposition, owing to the contingencies just mentioned on the part of the female; and also on some depending on the male; the penis being either so long as to reach beyond the mouth of the uterus, or the urethra so imperfect in its continuance along the penis, as not to reach beyond the labia. We have abundant examples of the former among blacks; and of the latter, *Morgagni** gives us some memorable instances.

3dly. Because the male organs do not possess sufficient power, when exerted even to their greatest degree, and when free from all restraint, to effect this purpose; but their power is constantly diminished, by the vagina for the most part surrounding and embracing the penis pretty firmly

* Morgagni de causis et sedibus morborum, Epis. xlvi. Art. 8, &c.

firmly throughout its whole length, and by the end of the penis coming in contact with some of the soft parts within the pelvis;* consequently, the impetus the semen derives from the parts destined to push it forward, must be very much abated; and its projectile force is not only thus nearly destroyed, but its direction is so altered, that it cannot effect a lodgement within the uterus.

4thly. Because the tenacity of the male semen is such, as renders its passage through the small aperture in the neck of the womb impossible, even by a power or force much superior to that which we may rationally suppose to reside in the male organs of generation.

5thly. Because the small aperture through which the semen must pass, is constantly lined, or rather filled, with a thick tenacious fluid, which alone would seem to offer an insuperable barrier to its progress, were the penis and os tincæ in the most favourable state of contact.†

Some, however, have been determined to overcome every difficulty that may be urged against this direct conveyance of the semen, by supposing the uterus possessed a power of admitting the penis by the opening of its mouth.

The admission, however, of this glaring stretch of probability, will not answer their purpose, unless they also shew us a power whereby the direction of the neck of the uterus may be constantly regulated; sometimes to advance it or make it recede; to elevate or depress it, as circumstances

* This especially happens in women who have had a number of children; for in them the uterus becomes habitually lower after each succeeding labour, so that their uteri lie for the most part just within the os externum: besides, many women are subject to a prolapsus of the womb so that this viscus occupies completely the vagina—yet impregnation takes place with them as readily, as with those who are not subject to this accident.

† Besides the reasons just mentioned, we may urge cases, where it was physically impossible for the semen to procure admission into the uterus, through its mouth, by the force exerted on it by the projecting organs. In one instance with which I am well acquainted, the opening of the urethra is not at the extremity of the penis, but under the glans and on one side of the frænum.—In another with which I am equally well acquainted, the impetus the semen receives, however powerful it may be, is effectually destroyed before it escapes from the canal, by a stricture in the urethra; a considerable time is therefore employed before the semen is discharged, and this is at last only effected guttatim. In both the above cases, the wives of these gentlemen bear children: nor is there the least room to suspect their fidelity. In these instances, how was the semen made to pass through the neck of the uterus?

stances may require: for we have already said, they were seldom or never in a state of apposition.

Besides, many cases of impregnation have taken place, where the penis never entered the vagina: a few of which we will relate.

Mauriceaux * mentions a case of a woman who conceived and was delivered of a child, although her hymen was not broken by coition.

Ruysch † relates a remarkable case of a woman being in labour whose hymen was entire, and against which the child's head pressed and prevented its delivery. He cautiously made an incision through it, and then perceived another thick membrane; this he also divided, and the woman was delivered.

Hildanus ‡ mentions a case somewhat similar to the two just quoted. He says, a young woman in Paris was married, but could not admit the embraces of her husband ; in consequence of which he sued for a divorce ; but the woman suspecting herself pregnant, was examined by several eminent surgeons, who found the entrance of the vagina shut by a strong, thick, callous membrane, in which were several small openings sufficient to allow of the discharge of menstrual blood. The membrane was divided, and by proper means kept open ; the husband was satisfied, and in six months the woman was safely delivered of a full grown child.

Harvey § says, he " knew a woman, who had all the interior part of the neck of her womb excoriated and torne, by a difficult and painful delivery: so that her time of lying-in being over, though she proved with child again afterward, yet not only the sides of the orifice of the neck of the womb near the nymphæ did close together, but all the whole cavity thereof, even to the inner orifice of the matrix, whereby there was no entrance even for a small probe, nor yet any egress to her usual fluxes. Hereupon the time of her delivery being arrived, the poor soul was lamentably tortured, and laying aside all expectation of being delivered, she resigned up her keys to her husband, and setting her affairs in order, she took leave of her friends. When, behold, beyond expectation, by the strong contest of a lusty child, the whole tract was forced open, and she was miraculously delivered," &c.

We

---

* Observat. 489. † Tom. 1. Observat. 22. ‡ Centuria III. Observat. lx.

§ Harvey Exercit. lxxiii. page 492.

We shall now add another remarkable fact from the same author. *

"The queen," says he, "had an exceeding white mare, excellently shaped, presented unto her: whose genital parts (lest by going to horse shee might endanger the beauty of her proportions, and become unfit for use) were, as the custome is, locked up all with iron rings. Notwithstanding which, this mare (by what accident I cannot tell, nor could the groomes inform me) was made big with foale; and at last, when they feared no such matter, she foaled by night, and the foale was found alive next morning by the mare's side."

We might easily multiply instances of the like kind, but these we trust will be sufficient to prove that conception has taken place where the hymen was entire, and consequently, where the penis did not enter the vagina to eject semen into the uterus, to form of itself a fœtus, according to the opinion of one set of theorists; to mix with the female semen as taught by a second; to moisten the womb, and by its aura impregnate the ovum, agreeably to a third; nor to travel through the Fallopian tubes to the ovaria in conformity with a fourth.

Besides, Harvey and De Graaf dissected animals at almost every period after coition, for the express purpose of discovering the semen, but were never able to detect the smallest vestige of it in the uterus in any one instance.

We are, however, well aware that Ruysch has asserted, in the most unequivocal manner, that he found the semen in its gross white state in one of the Fallopian tubes of a woman, who died very soon after, or during the act of coition.—But we conceive that this able anatomist must have been deceived as to the nature of the substance he found in the tube, and that it was not really semen: our reasons for thinking so are, first, that the semen after it has escaped from the penis, very quickly loses its albuminous appearance, and becomes as thin and as transparent as water. Secondly, if it be even admitted the semen has effected a lodgment within the uterus, what power exists there, to transport it in its original form to the Fallopian tubes? we know of no such power.

It may, however be urged, that the Fallopian tubes have the power of absorbing, and by this means would be able to take up the semen, and consequently, it might be found in them.

* Harvey, loc. cit.

But several important objections may be made to this opinion. First, How will the openings or mouths, if you please to call them so, of the tubes come in contact with the semen, or, in other words, how will the semen get to them, since it must occupy the lower parts of the uterus, and consequently be at least an inch from them? Secondly, the structure of the tubes is such, as forbids us to suppose absorption to be a part of their use. Thirdly, it would be assigning two offices to them, diametrically opposite to each other; first, to absorb and convey the semen to the ovaria; then to seize the impregnated ovum or ova, and carry it or them to the uterus. Need we say this is absurd? we have no analogy in the human body that we are acquainted with to support it.

We are therefore inclined to think, nay we are certain, that Ruysch was mistaken; some alteration in the natural secretion of the parts was mistaken for semen; this was nowise difficult for him to do, as he had a particular theory to support—and more especially, as this supposed discovery made so much for it. It is not merely speculative, when we say that some change in the natural secretion of the parts may have been mistaken for semen; for we have the testimony of Morgagni on our side. He tells us he has seen similar appearances in several instances in virgins and others, who had been subject during their lives to leucorrhœa. Ruysch's subject, though not a virgin, may have yet been troubled with this complaint.*

After having thus, we believe, rendered it more than probable that the semen never passes into the uterus, and in doing this, removed the objections founded on the contrary belief, to the possibility of superfœtation; let us proceed and see how we can support the idea of its taking place, when absorption from the vagina is admitted as the means, by which the male semen is applied to the ovaria.

This absorption may be effected in one of two ways; first, either by the common absorbents of the vagina taking up the semen, and going the route of circulation; or secondly, by a particular set of vessels which we shall call seminal absorbents, and which have a direct communication with the ovaria. We are inclined to believe it to be in the latter way; as it would seem to agree better with the general

---

* Morgagni indeed expressly tells us, when speaking of the natural secretion of the Fallopian tubes, that it had been mistaken by some for the semen virile.

*See Morgagni, Epist.* xxvi. *Art.* 13.

general simplicity of nature. No one to be sure has ever demonstrated these vessels (or as far as we know intimated a belief of them:) but this does not do away their existence, or invalidate our opinion of them. No one has yet ever shewn the lymphatics of the brain; yet it is admitted on all sides they exist; no one has ever traced them on the amnion; yet there is every reason for supposing them plentifully spread upon it; no one has ever followed them into the substance of bones, yet we have abundant proof of their constituting a part of them; no one has ever developed the muscular fibres of the uterus, yet the phenomena of labour puts it out of all doubt that it possesses them.

We shall therefore, nowithstanding we cannot demonstrate them, take it for granted they exist. We suppose them situated just within the vagina; some may be even external to it, and just within the labia; most probably they are in some instances pretty abundant here, as we see conception taking place when the semen could only have been applied to these parts. After the semen has been thrown from the penis into the vagina, it is confined there a longer or shorter time by means of the rugæ; these rugæ answer a double purpose, first, they serve to retain the semen that it may liquefy and more easily spread over the surface of the vagina; and, secondly, by their means a much larger surface is offered to be absorbed from. It is more than probable that these are the real uses of the rugæ. They may perhaps contribute in a degree to increase venereal pleasure, but this is certainly not their only use as some have imagined; for the doe, according to Harvey, has them in abundance; and he affirms, she always takes the male with reluctance and seeming pain. Moreover, we see immodest women enjoying the venereal congress, when their vaginas, from the long continuance of their debilitating habits, have the rugæ destroyed.

It may be asked, if there be this particular set of vessels within the vagina, for the express purpose of taking up the semen, why do they not also absorb the matter of gonorrhœa or lues, and thus produce the destruction of the ovaria, by conveying it to them? To this we might answer, that, such may be their economy or dispositions, that they are only roused to absorption by their own particular stimulus, namely, the male semen.

This arrangement is not unique; we have many instances of this kind in the animal system; thus, light admitted to the tongue produces no sensation; yet let fall upon the eye, powerfully affects it; the vibration of a musical

 chord, chord, or the tones of a flute, induce no change on the eye; but the ear is instantly influenced by them. But perhaps a more striking and just example may be taken from the economy of the lacteals of the intestines; they refuse admission to the excrementitious parts of our food, or in other words are only excited to action by their own proper stimulus, namely the chyle. It perhaps may be objected here, that various other substances are taken up by them besides chyle, such as the colouring matter of madder, mercury, &c. But we must recollect, that mercury never has been detected in the circulatory system; and Dr. Physick's experiments * go very far to prove it never is taken up. As to some other substances, we grant they may be, but must believe that they either are not in sufficient quantity or quality to make the chyle lose its peculiar stimulus. Nay, perhaps the arteries and veins may be justly considered in point; as we think it more than probable that no other fluid than blood would influence them to carry on the circulation. And we have arrived almost to a certainty, that no fluid save the male semen, will influence the ovaria so as to produce the phenomenon of a conception. It is true there are instances upon record, of hair and teeth being found in the ovaria of virgins, which might seem to contradict this belief; Dr. Baillie † and others furnish us with examples of this kind; but in these cases we agree with the Doctor that they are not the produce of conception; since, agreeably to Ruysch, ‡ they have also been found in a man's stomach; if they are thus accidentally produced, they may with as little surprise be formed in the ovaria as elsewhere: we therefore cannot admit them as exceptions to this last position.

Since then we know, that certain parts of the body obey only certain or specific stimuli, why may there not be a set of vessels that are obedient only to the stimulus of the male semen? for our own part we see no difficulty in admitting the idea.

Is not this opinion strengthened, by observing some women who have been barren with their first husbands, prolific with their second, and vice versa? The semen, in these unsuccessful instances, wanted that sufficient energy to call the seminal absorbents into action.

Besides,

---

* In a paper read before the Academy of Medicine.
† Morbid Anatomy, page 265.
‡ Ruysch, Tom. II. Aversar. Anatom. Decad. tert.

Besides, the very sudden effect which is sometimes produced by the male semen upon the female constitution, such as violent sickness, retchings, vomitings, nervous affections as they are termed, &c. will scarcely admit of explanation, on the supposition that it must go the tedious route of circulation before it arrives at the ovaria to produce its effects. And it will perhaps be difficult to conceive then, how it can be successfully applied to the ovum or ova, as it must still be contained in blood-vessels, whose sides are impervious in the living animal; whereas, the seminal absorbents most probably terminate on the ova, and thus, as soon as fit, will be subjected to the influence of the male semen whenever absorbed.

However, be this as it may, the male semen seems absolutely necessary to the production of the animal, and is in some way effectually applied to the ovum or ova, and thus produces the phenomena of impregnation.

Should there be but an ovum fit for the male influence, we shall have but one fœtus; if two, we shall have twins; and so on. But for the most part there is only one; nature kindly providing against the neglect that must necessarily arise from several being produced at a birth.

It would appear in general also, that a regular period elapses between the perfecting of each ovum; and hence we see women bearing children at stated intervals: for instance, every thirteen or eighteen months; every two, three, four, five, six, or seven years. Two, three, or four ova may chance to ripen (if we may so term it) at the same time; or, in other words may be in a condition to receive successfully the male influence; then we shall have, as we observed before, a corresponding number of children.

This law of perfecting the ova, however, is not immutable; there may sometimes happen a considerable variation in the term, but when in a condition may receive the stimulus of the male semen, and this may happen during the residence of a fœtus in utero; hence superfœtation. But the time which elapses, for the most part, is pretty uniform; and it would appear necessary also, that the first ovum or ova should be displaced before others can be perfected. This is a wise regulation of nature; otherwise, women who have lived long single, or been a long time deprived of commerce with man, would be subject to serious inconvenience; they would be liable to a litter of children. This rule obtains in other animals besides man.*

Let

* See Harvey, Spallanzani, &c.

Let us suppose now, a fœtus to be occupying the uterus; the woman to have a subsequent connection with her husband; the semen to be absorbed, and to meet with another ovum capable of being influenced by it; what will be the consequence? The ovum will be impregnated, and the ordinary changes will take place in the ovarium; the ovum will escape into the Fallopian tube, and through it pass to the uterus; here it will meet with a feeble resistance from the membranes which already line the uterus, and consequently cover the openings of the tube; this resistance will however be soon overcome, either by the ordinary efforts of the tube, or by the ovum resting unusually long, and beginning to develope, obliging the mouth of the tube to open, while it contracts with unusual violence behind, from the stimulus of distention, and thus forces it forward and displaces the slightly adhering membranes, and by this means will effect a lodgement in the uterus by the side of the other, where it will be as completely developed for the period of its stay, as though it had been placed there at the same instant with the other. It will have its own membranes, water, and placenta, having nothing in common with the other but its nidus.

In confirmation of the above doctrine, we beg leave to relate a couple of cases, complete we conceive in all their parts, to force the belief of the possibility of superfœtation; or, in other words, that the cases which we shall detail are really and bona fide cases of superfœtation.

Case I. On the 10th of October, 1799, at 5 o'clock P. M. I delivered a lady of a fine healthy boy after a labour of some hours. After a careful delivery of the placenta, I examined my patient by the vagina, and also by a hand upon the abdomen, to discover if there was another child, (for it was supposed by the lady's friends she was pregnant with twins) but could discover nothing like one. She was therefore put to bed, and enjoyed a sleep of several hours: she was roused from this at length by severe and regular pains; after they had continued some time, she felt something protruding from the vagina: this gave great alarm to her nurse and friends, and I was immediately sent for. When I arrived I found them in the greatest alarm, they supposing it was the uterus which had passed out. I immediately examined my patient, and found, instead of the uterus, an ovum complete. I extracted it carefully and entire. Upon opening the membranes, an embryo of between three and four months presented sented itself, looking fresh, and almost transparent; the funis large, white, and shining; the placenta healthy and entire; the blood on its maternal surface rather florid, a proof it had not long been detached from the uterus; the waters clear, abundant, and gelatinous; in a word, every thing looked as though the child had just parted with life. Those who are in the habit of seeing abortions, very readily distinguish between those which have just been deprived of life, and those which have parted with it a long time; this bore every mark of freshness; I was therefore much struck with its singularity.

The following considerations will, I think, establish beyond doubt that it was a case of superfœtation.

First, the absence of hæmorrhagy during the whole period of gestation; which would not have been the case, had the placenta been any time detached before the period of labour.

Secondly, the ovum having nothing in common with the full-grown fætus; on the contrary, it had its own membranes, water, placenta, &c.

Thirdly, the fresh and sound appearance of the ovum.

Fourthly, it having maintained its attachment to the uterus, after the birth of the other child; or at least it did not descend, so as to be discoverable by a careful examination by the vagina and otherwise, which renders its attachment more than probable, since this must and would have happened by the common tonic contraction of the uterus* after the birth of the other child and placenta; and that the uterus did contract is certain, as no hæmorrhage followed the extraction of the placenta.

Case II. A white woman, servant to Mr. H. of Abington township, Montgomery county, was delivered about five and twenty years since of twins, one of which was perfectly white, the other perfectly black. When I resided in that neighbourhood I was in the habit of seeing almost daily, and also had frequent conversations with Mrs. H. respecting them. She was present at their birth, so that no possible deception could have been practised respecting them. The white girl is delicate, fair skinned, light haired, and blue eyed, and is said to very much resemble

* By tonic contraction, we mean that regular and constant contraction whereby the uterus is reduced to its original size, after the distending causes are removed.

semble the mother. The other has all the characterizing marks of the African; short of stature, flat, broad nosed, thick lipped, woolly headed, flat footed, and projecting heels; she is said to resemble a negro they had on the farm, but with whom the mother never would acknowledge an intimacy; but of this there was no doubt, as he and the white man with whom her connexion was detected, both ran from the neighbourhood so soon as it was known the girl was with child.

We might produce other instances of superfœtation from the most respectable authorities, such as Aristotle, Harvey, &c. but suppose the above two sufficient, as it ought perhaps to be more a matter of surprise, why it does not more frequently take place, than that it should occasionally happen; as its occurrence or non-occurrence entirely depends on the contingency of the sooner or later arriving at maturity of the ova, and the absorption of semen.

